# Neurodivergent intersubjectivity: Distinctive features of how autistic people create shared understanding

**DOI:** 10.1177/1362361318785172

**Published:** 2018-08-03

**Authors:** Brett Heasman, Alex Gillespie

**Affiliations:** Department of Psychological and Behavioural Science, London School of Economics and Political Science, London, UK

**Keywords:** double empathy, friendships, intersubjectivity, neurodivergence, neurodiversity, norms, qualitative research, social interaction, video gaming, within-interaction variation

## Abstract

Autistic people are neurologically divergent, yet approaches to studying autism are framed by neurotypical definitions of being social. Using the concept of intersubjectivity, which conceptualises a variety of ways of socially relating, we investigate distinctive features of how autistic people build social understanding. A total of 30 members of a charity supporting adults with autism were video-recorded during a social activity they enjoyed, namely collaborative video gaming. Mapping the coherence, affect and symmetry of each conversational turn revealed shifting patterns of intersubjectivity within each interaction. Focussing on clusters of consistent and fragmented turns led us to identify two features of neurodivergent intersubjectivity: a generous assumption of common ground that, when understood, led to rapid rapport, and, when not understood, resulted in potentially disruptive utterances; and a low demand for coordination that ameliorated many challenges associated with disruptive turns. Our findings suggest that neurodivergent intersubjectivity reveals potential for unconventional forms of social relating and that a within-interaction analysis is a viable methodology for exploring neurodivergent communication. Future research should examine the varieties of neurodivergent intersubjectivity, with associated problems and potentials, and how those forms of intersubjectivity can be enabled to flourish, particularly in autistic-to-neurotypical encounters.

## Introduction

Autistic people are neurologically divergent, yet methods for investigating autistic sociality tend to assume neurotypical definitions of being social. Comparative design often results in autistic behaviour being interpreted as a deficit, rather than a difference, from neurotypical benchmarks (Kapp et al., 2013). Likewise, ethnographic research focuses heavily on autistic-to-neurotypical interactions which take place against the cultural backdrop of neurotypical norms and expectations (Heasman and Gillespie, 2017; Kremer-Sadlik, 2004; Ochs, 2015). Thus a methodological and empirical gap exists in understanding how autistic people relate to one another socially outside of conventionalised norms, which is important given reports from autistic people on how it is easier to relate to other autistic individuals precisely because of an absence of social protocol (Chown, 2014; Dekker, 1999).

We investigate interactions between 30 members of a charity supporting young autistic adults to identify the features of neurodivergent intersubjectivity evident in naturally occurring activities. Intersubjectivity was selected as an analytical framework, since it is suited to investigating diverse forms of socially relating, as evident in how autistic people relate to each other (Dant, 2015; De Jaegher et al., 2017; Samaritter and Payne, 2013). Using a systematic framework for identifying the shifting patterns of intersubjectivity in each interaction, we sought to map within-interaction variability and examine the features of such interactions.

### Intersubjectivity, neurodivergence and autism

Intersubjectivity is the process whereby people come together to create understanding (Gillespie and Cornish, 2010). Building intersubjectivity depends on the social situations, groups, norms and cultures encountered and the creative ways in which people play with imagined perspectives and cultural resources in their everyday sensemaking (Gillespie and Zittoun, 2010). Intersubjectivity differs from coordination, in that coordination focuses on consensus, whereas intersubjectivity characterises the diversity of ways people create shared understanding. For example, an interlocutor may share information that is not reciprocated or acknolwedged by another interlocutor in the next conversational turn. This may be deemed a failure to coordinate but equally count as a moment of intersubjectivity because it is an attempt to bridge ‘subjectivities’, an act which may be reciprocated, or become useful, at a later stage of the interaction. Thus when examining interactions, it is important to explore how interlocutors create possiblities for coordination, even if it is not consistently reciprocated immediately or if the process by which it is achieved is non-conventional.

Studies of intersubjectivity in autism have been primarily based on autistic-to-neurotypical interactions. These have highlighted difficulties such as shared intentionality (Tomasello et al., 2005) and reciprocating non-verbal cues (García-Pérez et al., 2007; Hobson and Lee, 1998). However, autistic divergence from the neurotypical norm for interacting (i.e. neurodivergent behaviour) can result in a gap in mutual understanding which makes empathy (Milton, 2012), perspective-taking (Heasman and Gillespie, 2017; Sheppard et al., 2016), and social perception (Sasson et al., 2017; Sasson and Morrison, 2017) difficult for both parties. This two-way misunderstanding has been termed the ‘double empathy problem’ (Milton, 2012), and it highlights the dangers of interpreting neurodivergent behaviour on neurotypical terms. Moreover, autistic interactions may be optimised differently across situations and groups (Bottema-Beutel, 2017; Ochs et al., 2004; Ochs and Solomon, 2010). Thus, although autistic people experience lifelong difficulties in social interaction, different contextual features of interactions can help to extend or limit possibilities for intersubjectivity, and such features need to be understood on their own terms outside of the application of normative criteria.

### Autism and video games

The aim of our study is to understand the features of neurodivergent intersubjectivity that sustain autistic-to-autistic interactions when shared experience, background knowledge and norms are arguably optimal. Accordingly, we recorded video game interactions between co-present autistic members of a charity supporting autistic adults because this was the most popular social activity in the charity and was thus suited to studying neurodivergent intersubjectivity on its own terms. Our overarching question is: what are the features of neurodivergent intersubjectivity observed in autistic-to-autistic interactions during collaborative video gaming?

Video games are popular among autistic people in general (Kuo et al., 2014; Mazurek et al., 2015) and among our participants specifically. Video games encourage active participation in the achievement of goals, and can be played across a variety of devices including dedicated consoles, computers and mobile phones. Video games, like all games, have a social basis (Gillespie et al., 2018); with sociality varying according to game format (e.g. single player versus multiplayer), game content (abstract puzzles versus virtual characters/terrains) and the context in which the games take place (solitary gaming versus collaborative public gaming) (Gentile, 2011). Our study involved players collaborating together on predominantly multiplayer games involving virtual social worlds and characters and thus entailed a highly social environment.

### Approach of the study

Intersubjectivity covers the variety of ways of socially relating to another. For example, it could take place across minds through language (Schegloff, 1992) and bodies through action (Hobson and Lee, 1998); it can also be contextually shaped by the types of activity undertaken (Linell, 2009) and occur across different timescales (De Jaegher et al., 2013). For the purposes of this study, we operationalised intersubjectivity by focussing on observable coordination in language. We reviewed existing interactional frameworks to identify the observable properties of intersubjectivity. Since such frameworks have been based on neurotypical interactions, our challenge was to mitigate the impact of applying normative criteria to our data. To achieve this, we avoided prescriptive categories (e.g. good or bad behaviour) in favour of descriptive categories which described a within-interaction change in dynamic. For example, our review identified three core aspects of intersubjectivity: (1) coherence (Linell et al., 1988; Roter and Larson, 2002) which describes the logical alignment from one conversational turn to the next, (2) affect (Bales, 1999; Nelson et al., 2016; Roter and Larson, 2002) which describes the emotional harmony between turns and (3) symmetry, which describes the alignment of conversational turns in terms of assertiveness/submissiveness (Angus et al., 2012; Bales, 1999; Linell et al., 1988).

Our study proceeded systematically through two steps. First, we mapped out the temporal shifts in intersubjectivity within each interaction to identify sequences that are either consistent or fragmented in terms of coherence, reciprocation of affect and symmetry. Second, we analysed these sequences qualitatively to explore how social coordination is achieved, which led us to identify two features of neurodivergent intersubjectivity.

## Method

### Participants

Observation took place at a charity supporting adults with autism. Available activities included music, strategy games, art, pool, Lego and, the most popular, video games. All 30 participants were members of the charity, had either a confirmed diagnosis of autism (n = 24) or had been referred for assessment by a medical professional (n = 6) and had no history of significant verbal comprehension or intellectual challenges. Inclusion criteria was broad due to challenges associated with consistent diagnosis (Liptak et al., 2008; Turowetz, 2015) and extensive delay in assessment (+2 years in local area), thus participants referred for assessment by a medical professional, but still awaiting diagnosis, were included. Our sample included a gender bias towards males (25:5) with a mean age of 23.6 (range: 16–34) years.

### Materials

The study used a dedicated room with an Xbox One games console, two controllers and a large LCD TV screen. Current popular games in the UK chart were made available to the participants: *Assassin’s Creed: Syndicate* (1-player), *Call of Duty: Advanced Warfare* (2-player), *Halo* (2-player), *FIFA 14* (2-player), *Forza Motorsport 5* (2-player), *GTA V* (1-player) and *Lego Batman 2* (2-player). For single-player games, two participants took it in turns to control the avatar, the decision of which occurred naturally without intervention from the researcher. In such cases the other player provided advice and commentary in periods without the controller. Two cameras captured (1) the participants’ activity and (2) the video screen. All interactions were fully transcribed (see Supplementary file 1 for transcription notation).

### Procedure

Ethical approval was granted by the researchers’ university and the charity where the research was conducted. Participants were made aware of the nature of the observation, why it was taking place and how the data would be stored, anonymised and analysed. Details of the observation were sign-posted at the entrance to the room with charity staff and the researcher available to answer questions. Prior to each video-recorded interaction, the researcher ensured participants understood the video-recording and consent criteria and made their right to withdraw at any time clear. Initially some participants were curious during the explanation of the study ‘about social interactions’, and discussed the recording equipment while games were loading, which could potentially result in altered behaviour through increased self-consciousness. However, all participants quickly became absorbed by the activity of gaming, and their attention very rarely returned to the recording equipment, shown by their lack of verbal reference or visual attention (i.e. looking at the equipment).

In total, 20 sessions were recorded involving 30 participants, with 10 participants taking part in more than one session (Table 1). No session involved a duplicated set of participants. The researcher was present in the interactions to assist with any equipment issues and contributed to the conversation at the beginning (during set-up) and at the end (concluding the session). The researcher was available to answer questions when prompted by participants and was seated adjacent to both the gamers and the TV screen out of the gamers’ line of sight.

**Table 1.** Summary of interactions and games played.

Interaction	Players	Game format	Game content	Duration (min)	Words	Words per minute	Turns
1	4	2-player	Lego Batman	26	3043	117.04	302
2	2	1-player	Assassin’s Creed	58	4790	82.59	454
3	2	2-player	Halo	58	5665	97.67	704
4	4	2-player	Call of Duty/Forza	48	5350	111.46	427
5	3	1-player	GTA	27	3783	140.11	348
6	2	1/2-player	GTA/Call of Duty	57	6545	114.82	797
7	3	2-player	Forza	34	1911	56.21	205
8	2	2-player	Forza/Lego Batman	18	659	36.61	68
9	2	2-player	Call of Duty	35	4392	125.49	300
10	2	2-player	FIFA	51	3327	65.24	342
11	2	2-player	Forza	54	1997	36.98	231
12	2	2-player	Call of Duty	35	3429	97.97	301
13	4	2-player	FIFA	38	1735	45.66	193
14	4	2-player	Call of Duty	33	3018	91.45	286
15	2	2-player	Call of Duty	30	2207	73.57	110
16	2	2-player	FIFA	26	1402	53.92	135
17	5	1-player	Assassin’s Creed	32	3460	108.13	258
18	2	1-player	Assassin’s Creed	33	4465	135.30	315
19	2	1-player	Assassin’s Creed	54	4544	84.15	255
20	4	2-player	Lego Batman	20	3098	154.90	335
Total				767	68,820		6366
Average				38.35	3441	91.46	318.3

### Process of analysis

To analyse the transcript, we operationalised a conversational turn as the period from which a speaker initiates an utterance to when the utterance concludes and another speaker assumes control (Sandvik et al., 2002). To understand broad patterns of within-interaction variability, each turn was scored, on the three dimensions of intersubjectivity, on a scale of −1 showing fragmentation with prior turn, to +1 showing alignment (in cases of affect, harmony) with prior turn. A score of 0 represented turns that were ambiguous, unclear, or failed to meet any explicit criteria for coherence, affect or symmetry (see Supplementary file 2).

We operationalised the three dimensions in the following way to understand within-interaction variability. Coherence focussed on topicality and was scored in terms of how a turn is part of the sequential organisation of interaction. For example, question and answer sequences (known as adjacency pairs) would have the answer turn scored as +1 (thus showing it is in alignment with the prior turn), whereas interrupting to change topic would result in a turn scored as −1 (in misalignment with the prior turn).

Affect focussed on emotion displayed. Since we were examining only observable displays of affect, many turns were ambiguous to rate (resulting in a 0 score), thus computing alignment between turns would result in a disproportionately high score, (i.e. consecutive zeros would count as strong alignment). We therefore operationalised affect in terms of emotional harmony to understand within-interaction variability. Criteria for scoring affect was very conservative, including only clearly positive and clearly negative turns (e.g. laughing, complimenting = positive (+) 1 scores; criticising, complaining = negative (−1) scores).

Finally, symmetry focussed on how assertive/submissive a turn was relative to the prior turn. Since evident in every turn, symmetry was operationalised similarly to coherence, thus if both speakers were quiet, or both ebullient, there was symmetry in terms of +1 scores.

Inter-rater tests were conducted with an autistic adult with a confirmed diagnosis of Asperger’s syndrome. Two raters (who were not participants) were first shown the application of the rating framework to a transcript, with the researcher answering questions. One rater discontinued because they stated they were bored with the task and did not provide any feedback about the framework. A second rater enjoyed the task and provided feedback about the framework. The main discussion points were how to rate very short turns which may be shaped by the prior context. For example, “OK”, could be scored high for coherence (+1) if the prior turn is an instruction (e.g. “we will restart”) or scored as ambiguous (0) if the prior turn is an open question (e.g. “What track should we race on?”) or scored as fragmented (−1) if spoken to interrupt the prior speaker and change topic. In two separate sessions, the rater randomly selected two transcripts to rate, completing 316 turns.

To make the interactions of comparable length, we analysed all interactions up to the 300th turn and, after rating all turns, researcher turns were removed from the analysis, thus capturing how autistic participants responded to any interactions with the researcher but preventing the researcher from influencing scores.

To build an overview of the data sample and understand how interactions compared with each other, turns were categorised as consistent (involving +1 with no −1 scores), fragmented (involving −1 with no +1 scores) and mixed (involving +1 and −1 scores, as well as ambiguous turns involving only 0 scores). Clusters of three consecutive turn types highlighted areas for in-depth analysis, since three turns is the minimum unit for co-constructing knowledge (Schegloff, 1992).

To understand within-interaction variability, we mapped interactions longitudinally using line graphs and the ratings given for each intersubjective dimension. Initially, this results in a noisy graph; therefore, to smooth out noise and identify the trends, we took a moving average of each intersubjective score. Averages of ratings for conversational turns have been used in interaction frameworks before to benchmark performance between interactions (Linell et al., 1988). We used a moving average to facilitate our goal of understanding within-interaction variability. Through trial and error, we found that a 20-turn moving average provided an optimal resolution for identifying overarching peaks and troughs in intersubjectivity.

Qualitative analysis proceeded by comparing consistent and fragmented clusters of dialogue with intersubjective scores to identify ‘enabling’ moments, (i.e. an observable increase in subsequent turns of one of the three dimensions of intersubjectivity (coherence, affect or symmetry)). An abductive process (Tavory and Timmermans, 2014) involved iteratively exploring (Neale, 2016) the intersubjective features that could help to explain within-interaction variability, with clusters expanded to include relevant context. Abductive processes involve an interpretive step guided by the surprising phenomenon observed and the explanatory scope of subsequent hypotheses generated about the data.

## Results

### Mapping dimensions of intersubjectivity

**Figure 1.**
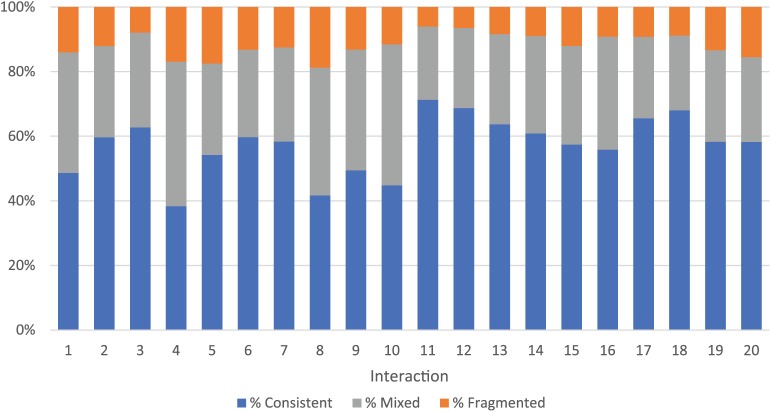
Percentage of interaction involving consistent and fragmented coordination turns.

Inter-rater reliability analysis using Cohen’s Kappa for each intersubjective dimension yielded moderate to high levels of reliability (coherence = 0.592 (p < 0.001), 95% confidence interval (CI) = 0.512−0.672; affect = 0.786 (p < 0.001), 95% CI = 0.708−0.864; symmetry = 0.583 (p < 0.001), 95% CI = 0.497−0.669). Across interactions, there was a relatively stable pattern in terms of overall percentage of turns that were either consistent (mean = 57%) or fragmented (mean = 12%) with the prior turn (See Figure 1). All dyads successfully coordinated during gameplay; but one dyad had an argument (interaction 4) and another had a lack of communicative responsiveness (interaction 8).

Mean scores of dimensions of intersubjectivity showed variation within interactions (see Table 3 standard deviations, in Supplementary file 3). Scores were based on 20-turn moving averages, thus a mean score of +1 would indicate that dyads were in perfect alignment, and a mean score of −1 would indicate that they were in complete misalignment, over 20 turns. All dyads had 20 turns in which there was at least +0.4 alignment across intersubjective dimensions, and also 20 turns in which there was at most only +0.1 alignment across dimensions (see Table 4, in Supplementary file 3).

Autistic interactions have been characterised as overtly logical (Hermelin and O’Connor, 1985), but we also found displays of positive affect to be common (mean across interactions = +0.24, SD = 0.54), with laughter, encouragement and joking widespread (e.g. interaction 15). It was also possible for coordination to involve high symmetry (mean = +0.34, SD = 0.62) despite low coherence and affect (e.g. interaction 10), such as when players vented their frustration at their virtual avatars.

**Figure 2.**
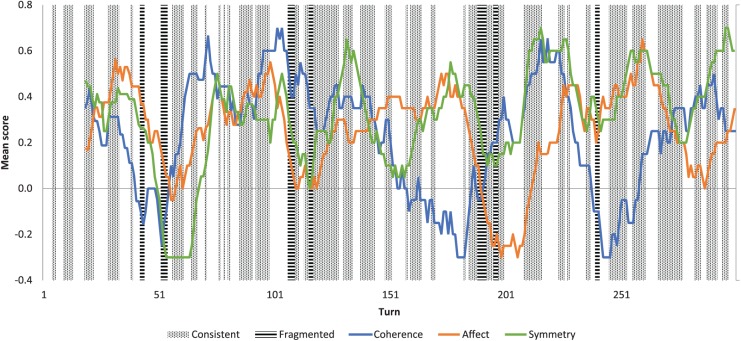
Example interaction, with 20-turn moving average of intersubjective dimensions and 3-turn clusters of consistent and fragmented moments.

Sequential mapping of interactions (Figure 2) highlighted two key phenomena: (1) rapid shifting between consistent and fragmented moments and (2) divergence between intersubjective dimensions. For instance, in Figure 2, rapid changes in consistency reflected shifting interactional trajectories, for example prior to turn 60 symmetry is low as one player dominates dialogue but switches to being high when two new players enter the room and introduce themselves (turns 50–100), leading to tighter turn-taking (increasing coherence and symmetry) and politeness scripts (increasing affect). Likewise, turns 160–185 have high affect and symmetry but low coherence because both players are sharing stock phrases of characters from the *DC comics* universe, while turns 205–220 have high coherence and symmetry because players have mutually identified a cooperative in-game task but are criticising each other’s efforts (hence low affect).

Mapping the dimensions of intersubjectivity raised questions, namely how do the interactions lead to rapid shifting between consistent and fragmented intersubjectivity and how is it that the three dimensions of intersubjectivity can diverge so sharply, such as when coherence is low, but affect and symmetry are high?

### Identifying features of neurodivergent intersubjectivity

To address questions of within-interaction variability, we analysed clusters of consistent and fragmented dialogue. An abductive process (Tavory and Timmermans, 2014) of comparing and contrasting different clusters explored features that explained patterns apparent in the data. Features explored included spontaneous voicing, self-directed speech, invitations for further speech and adjacency pairs responded/ignored. However, two features emerged as both potentially pervasive and consequential in explaining the patterns identified: (1) a generous assumption of common ground and (2) a low demand for coordination.

### Generous assumption of common ground

The rapid shifting between consistent and fragmented turns across interactions was associated with moments where participants made generous assumptions of common ground. Autistic difficulties in maintaining interactional trajectory are well documented, including low use of cooperative signals (Capps et al., 1998) and seemingly egocentric orientated speech (Frith and De Vignemont, 2005). In our data, we had many comparable cases of sudden and specific topic shifts. This often manifested in moments where one player spontaneously adopted the voices of fictional characters without signalling their origin to the other player or following up to ensure mutual understanding (see Supplementary file 4). Such instances may result from the potential for language to be experiential, that is, the act of speaking and hearing words is a constituent part of experiencing an object (Sterponi et al., 2014). Yet vocalising such perspectives assumes to some degree a level of common ground; when the voices related to characters in the game, they were more likely to be reciprocated (70%) than when they related to fictional characters beyond the immediate context (52%). In our data, sometimes, these generous assumptions of common ground could fragment coherence, but other times it could spark creative, productive and affective passages of dialogue:

Example 1.

Voicing leading to humour, interaction 02. Both participants with confirmed diagnosis. Change in 20-turn average score between turns 93–97, coherence = −0.15, affect = +0.20 and symmetry = −0.15.

**Table:** 

93	Daniel:	I need more knives because they are not knives ‘this is a kni:fe’ ((*Australian accent*))	
94	Max:	Ha ‘no that’s a spoon’ ‘Oh you’ve played knifey spoony before?’ ((*Australian accent*))	←
95	Daniel:	Yea I love that show	←
96	Max:	‘I like the choo choo’ ((*possible parody of a train spotter*))	
97	Daniel:	(5.0) Right I’ve got to get in here	

In Example 1, Daniel and Max are playing *Assassin’s Creed*, and Daniel is trying to select an optimal weapon from his arsenal. He observes his current knife is inadequate, leading him to voice “this is a knife” (93), a cultural reference to the film *Crocodile Dundee* (Cornell, 1986), where the protagonist produces a large knife in response to being robbed. Max recognises the reference shown by his laughter and then responds with two further voices ‘no that’s a spoon’ ‘Oh you’ve played knifey spoony before?’ (94) which is dialogue from *The Simpsons* cartoon series (Oakley et al., 1995), where Bart goes to Australia and encounters a character parodying Crocodile Dundee. Max assumes Daniel will understand the nested cultural reference, which he does (95) by referring to a “show” and not a film. Thus, in this instance, the generous assumption of common ground produces highly coherent, affective and symmetric coordination – but without knowing the cultural references, it might appear fragmented. In the following line however, Max continues with another voice (96). Daniel ignores this turn, and instead refocuses their attention on the task in hand, highlighting the varied nature of coordinating through voices in the dialogue.

In Example 2, James and Bruce are playing *Call of Duty* when a new enemy appears, a spaceship with a powerful laser.

Example 2.

Shared language, interaction 09. Both participants with confirmed diagnosis. Change in 20-turn average score between turns 92–105, coherence = +0.30, affect = +0.35 and symmetry = 0.

**Table:** 

92	James:	Who the fuck is this?	
93	Bruce:	ah ((*laughs*)) jumped into it	
94	James:	‘THEY HAVE SUMMONED THE RED CIRCLE OF DOOM’	←
95	Bruce:	It’s Tron (.) ‘All hail Tron (.) A:h’	←
96	James:	‘Hail!’	
97	Bruce:	You got to lay down and worship Tron (.) ‘A:h worship Tron’	
…			
103	Bruce:	Where the fuck are ya? oh you are there (1.0) fucking hell I think just being near you was hurting me there (.) Oh no there’s a =	
104	James:	= It’s Tron!	←
105	Bruce:	<Yea you wanna be back> (4.0) I was going to say it’s probably best if you just come (.) down here and stand by this entrance down here since you are that big guy (.) ’cause you really need to (.) you need an open area to be fighting in	

Example 2 highlights how James and Bruce create new shared language to index the ambiguous spaceship laser and the corresponding action to take. James’ initial problematisation (92) is not responded to, as Bruce is distracted by damage to his avatar’s health from jumping into the laser (93). James nonetheless adopts a dramatic narrator’s voice in a second attempt to make sense of the threat (94). Bruce reciprocates, not coherently, but stylistically through a voice which indexes the *Tron* (Kushner and Lisberger, 1982) film franchise (95). Turns 96 and 97 reciprocate understanding of the Tron reference as players parody worshipping Tron as a god. When the spaceship appears later (103), James identifies it using Bruce’s original Tron reference, which prompts Bruce to provide advice based on his prior experience (105). Thus in playing with voices, players are able to develop shared language on the basis of their shared cultural resources which allows them to creatively index and orientate to novel problems.

Examples 1 and 2 illustrate how a generous assumption of common ground, such as by sharing very specific voices, can lead to rapid rapport, with very closely aligned intersubjectivity. However, in moments when a generous assumption of common ground does not work, it can create discoordination and appear egocentric.

### Low demand for coordination

Research on autistic interactions has highlighted disconnect in terms of coherence (Tager-Flusberg and Anderson, 1991), pragmatics (Baltaxe, 1977; Volden, 1997) and detecting sociocultural cues (Kremer-Sadlik, 2004). In our data, there were many instances of small-scale misunderstandings resulting from ignored turns, parallel dialogue (independent conversation threads maintained over several turns) and misinterpretations (misreading the pragmatic/emotional context of the prior turn). However, these were not always problematic precisely because participants demonstrated a low coordination threshold and were able to move on quickly from disconnected and disruptive turns.

Example 3 illustrates an interaction between Billy (who is experienced at first-person shooter games) and Susan (who is less experienced) as they play *Call of Duty*. Billy is showing Susan the controls:

Example 3.

Misinterpreting prior turn, interaction 04. One participant with confirmed diagnosis, one participant awaiting assessment. Change in 20-turn average score between turns 37–49, coherence = +0.25, affect = +0.50, symmetry = +0.15.

**Table:** 

37	Billy:	So that’s to shoot (.) e:r that’s to: like jump (.) <you know like> ((*moves hand upwards*)) (.) if you click that forward like (2.0) like that you go forward you click it side (.) jump you click it side you go (.) (*moves hand left and right and makes air movement sound)* <swft swft swft>	
38	Susan:	Oh right that sounds fair enough (.) easy.	←
39	Billy:	<all right> what was ‘D’? What was to swap the gun? You see I have stopped playing these games (.) I think it’s ‘X’	
40	Susan:	We will work it out	
41	Billy:	Oh I guess so (.) Oh yea I was going to lie to you (.) ‘THAT one’s to shoot’ ((*indicates an incorrect shooting button on the controller and smiles*))	←
42	Susan:	((*turns to look at Billy’s controller and is suddenly confused*)) Which one’s to shoot?	←
43	Billy:	((*suddenly confused*)) Like if so (.) show me again? ((*points to button on Susan’s controller. Game announces: ‘Bad guys heading your way. Help will not be arriving soon’*)) OK so (4.0) right I’m coming to you	←
44	Susan:	Where are you?	←
45	Billy:	Oh you are on my team apparently	
46	Susan:	Good	
47	Billy:	Yay (.) all right so I am right here (.) ‘Hello? Hello?’ (.) right ((*Susan shoots Billy*)) Hey (.) fuck off	←
48	Susan:	Ha ha sorry	
49	Billy:	What the hell’s wrong with you? ((*smiling*))	

Example 3 illustrates a misinterpretation of pragmatics by both players. In turn 38, knowledge about how to shoot is agreed upon, but Billy later reveals his plans to misdirect Susan (41). Susan initiates a ‘repair’ (Schegloff, 1992) because she does not recognise that Billy’s prior turn was said in jest (42). Billy then responds to the literal request from Susan, not recognising that she herself has misinterpreted his joke (43). Coordinating tightly seems to be a low priority, as illustrated in the ignored question in turn 44. Although coherence is low, affect and symmetry are high, thus the misunderstanding leads to greater certainty about the functions of the game controller during gameplay, as evidenced by Susan’s first action in the game, which is to shoot Billy (turn 47).

Example 4 involves David (who has the controller) and Mark who are working together to play *Assassin’s Creed*. To begin with, David, who is more familiar with the game, is interested in how the game has developed new features in comparison to previous games. Mark, being new to the game, is marvelling at the graphics:

Example 4.

Parallel dialogue, interaction 17. Both participants with confirmed diagnosis. Change in 20-turn average score between turns 119–139, coherence = −0.55, affect = +0.50 and symmetry = +0.30.

**Table:** 

119	David:	yea there are going to be shops	
120	Mark:	(2.0) I like really (.) yea they have put a lot of effort into this game	
121	David:	vehicle attacks =	
122	Mark:	= I look at the buildings and I think ‘my god’	
123	David:	(2.0) I know something that was like a big deal was vehicle (.) vehicle attacking (.) like you could pull up to an enemy stage coach jump across beat them up (.) I think that was a thing	
124	Mark:	(4.0) I wouldn’t even spend my time playing the game I would just be walking around (.) admiring the view	
125	David:	(2.0) Yea I was on Assassin’s Creed three and I just got so bored with the stealth I just wanted to blast through the story as quick as I could =	
126	Mark:	= Look at that! That Westminster Church is amazin	
127	David:	grappling hook (.) god I am really Batman now	
128	Mark:	(3.0) That is so: co:ol ((*in response to the scenery*)) (22.0) yea gotta see what it looks like (19.0) I was just admiring the detailing on the Westminster building	
129	David:	(4.0) the controls are a bit annoying sometimes you can’t exactly tell it what to do (19.0) Oh god I really want to see if I can just grapple over to there	
130	Mark:	Oh my god it makes me feel queasy (.) OH GOD	
131	David:	I never experience that with games I don’t think I get vertigo	←
132	Mark:	no don’t jump (.) can you imagine this on VR? You are up there but you actually feel as if you are up there?	
133	David:	(3.0) I know I would like =	
134	Mark:	= look at my hands (.) look at my hands sweat	
135	David:	have you ever watched ‘Jack Septic Eye’?	
136	Mark:	no	
137	David:	right he plays a lot of video VR games (.) and he has this fear of heights (.) like a proper fear of heights (.) but I have seen him play Spider Man Homecoming or like this little taster for it (.) and it was like really fun but he was terrified of the swinging	
138	Mark:	was he was he genuinely?	
139	David:	yea he was genuinely terrified from it	

Example 4 shows David and Mark cooperatively turn-taking about two separate topics (119–130). Mark is focussed on his embodied reaction to the game, his admiration (122) turning to nausea (130), while David’s focus on relating the game to past games (e.g. 125) develops into a concern about game controls (129). What is striking is how there is minimal coherence up until turn 131, yet both players are affectively engaged in expressing emotions of curiosity and excitement (high affect and symmetry). Eventually, their intersubjectivity becomes coherently orientated in turn 131 as David directly responds to Mark’s observation about feeling queasy, perhaps because their dialogue has converged around the topic of height. This initiates a sequence of reciprocated turns (131–133, 135–139) during which new knowledge about the relationship between vertigo and graphically intense games is established allowing the players to build rapport. Previous studies have observed the tendency for autistic children to drift between topics leading to ‘irrelevant’ responses (Loukusa et al., 2007). However, Example 4 highlights how this tendency is made unproblematic by the low demand for coordination; indeed, it allows the players to build rapport and knowledge, since they are free to drift between individual and cooperative ways of verbalising their relationships to the situation, even if to the neurotypical observer this process may appear disjointed to begin with.

### Complimentary intersubjective features

The examples analysed here have shown how a generous assumption of common ground and a low demand for coordination can have enabling outcomes as evidenced by their reciprocation and development in proceeding turns. In Example 5, Daniel and Max are interrupted by two new visitors, Graham and Alice. Graham introduces Alice who has never met Max or Daniel before. Following the introductions, Graham begins to initiate their exit from the room:

Example 5.

Complimentary neurodivergent intersubjectivity. All participants with confirmed diagnosis. Change in 20-turn average score between turns 74–98, coherence = +0.75, affect = +0.30 and symmetry = +0.35.

**Table:** 

74	Graham:	Well well thank you I hope we weren’t really disturbing?	
75	Max:	Na its ok don’t worry about it	
76	Graham:	Yea >I mean I mean< Alice are you thinking of hanging around or do you want to go out now that you =	
77	Alice:	= Erm I don’t mind	
78	Daniel:	[Yea show her around	
79	Max:	[Yea show her the music room	
80	Graham:	I have pretty much just done that. But (.) I know you used to play the violin but I (.) I mean I am not sure if you will enjoy anything else, but =	
81	Alice:	= Not really no	
82	Max:	>GET HER A VIOLIN AND PLAY THE< HALO THEME	←
83	Graham:	ᵒYea wellᵒ	
84	Max:	I’m not sure it’s the violin (.) or is it a cello?	
85	Alice:	I c’n (.) I can do the Skyrim theme	
86	Daniel:	Oh nice one!	
87	Max:	Nice!	
88	Daniel:	((*laughter*)) Very nice	
89	Max:	You have earned my respect	
90	Alice:	You guys are playing DC (.) as a Marvel fan I must leave	←
91	Max:	Yea this ain’t our choice I wanted to play Halo MasterChief collection of something (.) but that would take too long to install	
92	Alice:	I would like to point out that Batman is basically Tony Stark who wasn’t clever enough to build himself a suit	←
93	Daniel:	((*Daniel laughs*)) that sounds actually (.) viable	
94	Max:	That’s actually true (.) although you know on Batman’s behalf (.) I mean (.) come on he’s been around longer (.) has accomplished more (.) of course let’s face it Iron Man does look cooler	
95	Alice:	Very much cooler (.) Batman is a panzy	
96	Max:	Iron Man is just, it’s an >it’s an< awesome suit	
97	Alice:	Iron Man is here to chew bubble gum and kick ass and he’s all out of bubble gum	←
98	Max:	Yes (.) ((*nods*))	

In line 82, Max shouts loudly across the room at Alice (82). His instruction to Alice disrupts the script of exiting that Graham had initiated in turn 74. Graham’s unsure response (83) shows that he is not familiar with Max’s reference to music within a specific game. However, Alice connects with the cultural reference because she plays *Skyrim* and is thus part of the symbolic world of console gaming (85). Revealing this, ‘I c’n do the Skyrim theme’, creates mutually recognised common ground leading to rapport-building as Alice takes control with a series of epigrams (90, 92 and 97) that are familiar and appropriate within this sub-culture. Thus, the complimentary nature between the generous assumption of common ground (i.e. Max’s very specific sub-culture reference) and the low demand for coordination (i.e. Graham not picking up on the common ground and neither Graham nor Alice perturbed by being shouted at) unearths new intersubjective potential to engage socially, which otherwise would have been undiscovered.

## Discussion

This study explores autistic interactions through assessing within-interaction variability, but before discussing its implications, we give consideration to its methodology and limitations. We operationalised intersubjectivity in terms of coherence, affect and symmetry; however, alternative ways of operationalising intersubjectivity (e.g. different criteria, moving averages and interpretation of qualitative extracts) may lead to different results. For example, explicit features of language are only a partial window into how people relate to one another (e.g. silences and non-verbal communication have not been considered). Undoubtedly more features of neurodivergent intersubjectivity will be identified when studies include additional communicative features and contexts. The methodological contribution of this study is to show the utility of studying interactions in terms of within-interaction variation.

A challenge faced was how to interpret neurodivergent interactions outside of normative criteria, particularly when previous interaction frameworks are based on neurotypical data (e.g. doctor-patient interactions). To mitigate this, we selected only broad features of intersubjectivity, but further analyses may wish to consider more specific criteria, such as examining the structure and quality of ‘repair sequences’ in dialogue (Schegloff, 1992). We also conducted inter-rater reliability with an autistic rater. The authors recommend that future studies of autistic social interaction use autistic inter-rater reliability as a means of questioning neurotypical assumptions that may be embedded within the research.

Our sample is not representative of the diversity of people on the spectrum, given its gender bias, age range and focus on verbal competence, thus the findings are not indicative of all examples of neurodivergence. Future studies should examine neurodivergent intersubjectivity within different activities and cultures, given the extent to which interactions are shaped by context (Gernsbacher et al., 2017). Additional contexts will help to expand and refine the current rating framework and improve inter-rater reliability. Moreover, research is needed to systematically compare neurodivergent intersubjectivity with a neurotypical control group and other neurodivergent groups to understand whether the features of neurodivergent intersubjectivity observed here are generalisable to other contexts where neurotypical norms for interacting are not observed.

Mapping dimensions of intersubjectivity in interactions involving neurodivergent participants raised two questions, namely how do the observed interactions facilitate the rapid shifting between consistent and fragmented intersubjectivity, and how is it that the three dimensions of intersubjectivity can diverge so sharply? (e.g. low coherence, but high affect and symmetry). We observed two features of intersubjectivity that help to explain these phenomena.

First, a low demand for coordination could lead to fragmentation (e.g. players not coherently aligned), but it could also ameliorate many of the challenges associated with fragmented or potentially disruptive turns allowing players to swiftly move on from small-scale social misunderstandings (e.g. accidental other-initiated repair in Example 3; ignoring shouted turn in Example 5). Second, abrupt topic shifts, particularly through perspective-playing with characters from films, TV, music and imagined perspectives, could create new rich dialogue despite potentially fragmenting coherence. Everyday social exchanges take place upon a foundation of assumed common ground (Garfinkel, 1964). Indeed, intersubjectivity can never be known at the outset; it needs to be assumed to be achieved (Rommetveit, 1976). Accordingly, the generous assumptions of common ground made by neurodivergent participants allowed underlying sub-cultures to be identified, leading to the rapid construction of shared understanding, rapport and humour. When generous assumptions of common ground fail to result in reciprocated turns, it may appear egocentric to the outside observer, but when reciprocated, it can lead to increased affect, symmetry and coherence, creating a rich intersubjective space for shared understanding.

The generous assumption of common ground and the low demand for coordination are more than two isolated features; they potentially fit together into a functional system that allows rich forms of social relating which can explain how rapid changes in interaction dynamic are possible. It allows autistic participants to continually experiment with ways of relating to their situation incurring minimal detrimental impact to their social identity when references are not shared. It is the way that these two features fit together to allow distinctive ways of building shared meaning that we describe as a feature of neurodivergent intersubjectivity.

Our findings support previous research on the under-recognised ability of autistic peers to be motivated and able to manage interactions with one another (Brownlow et al., 2015; Ryan and Räisänen, 2008) and highlight the need to examine other contexts for autistic social interactions, particularly given the potential for the activity of gaming to support the features observed. For example, facing the screen and not each other circumvents the challenges of face-to-face communication that many autistic people experience (Parsons and Cobb, 2011). Affect scores may be improved through the motivating (Granic et al., 2014) and captivating (Ash, 2013) aspects of video games, while repetitive gaming can enhance learning and establish context (Squire, 2006), making assumptions of common ground easier to manage. Gaming also involved the integration of first-person (direct experience), second-person (talking to each other) and third-person (shared object of the screen) perspectives with frequent position-exchange of social roles (i.e. helper-receiver, attacker-defender and teacher-learner) allowing autistic participants to explore and play with perspectives that they might not otherwise have exposure to in other domains of social life (Wijnhoven et al., 2015). This shared focus may account for some of the flexibility participants demonstrated in changing topic and following implicit references, thus studying other contexts without an object of shared focus will help to illuminate more about the situational resources which support neurodivergent intersubjectivity.

Further studies may also explore neurodivergent intersubjectivity in cross-neurological contexts (e.g. job interviews) to understand the nature of the ‘double empathy problem’ (Heasman, 2017; Milton et al., 2018). Our findings reveal that neurodivergent interactions provide opportunities for rich intersubjectivity even when faced with severe fragmentation and raise questions as to whether neurotypical norms potentially limit this possibility because they interpret such fragmentation as failures needing to be addressed – thus limiting the potential of the conversation to move on. For example, the difficulties autistic people experience in indexing sociocultural meaning (Ochs and Solomon, 2010) are not so problematic when sociocultural conventions are relaxed because some autistic adults are able to delve into their own repositories of symbolic resources to generate localised meanings and develop mutual understanding. Likewise, fragmentation of the interaction coherence by attending to different aspects of the interactional context (Bottema-Beutel, 2017) is less of a problem when norms permit the spontaneous interchange of private and social speech. Thus our findings highlight how neurodivergent intersubjectivity can potentially create rich social interactions. Certainly, a first step to allowing neurodivergent intersubjectivity to flourish (or at least not be undermined) is to recognise it as having distinctive features that can be enabling.

## Supplementary Material

Supplementary material

Supplementary material

Supplementary material

Supplementary material

Supplementary material
