# Supplementary material for: Neurodivergent intersubjectivity: Distinctive features of how autistic people create shared understanding
Source: Autism. 2018 Aug 3;23(4):910–21. doi: 10.1177/1362361318785172 (PMC6512057; doi:10.1177/1362361318785172)
Supplement: Supplementary material [file AUT785172_Lay_Abstract.pdf]

Lay Abstract

autism

DOI: 10.1177/1362361318785172

Neurodivergent intersubjectivity: Distinctive features of how autistic people create shared understanding

Brett Heasman and Alex Gillespie

This study investigates interactions between autistic people to identify distinctive ways in which they create shared understanding. Thirty members of a charity supporting autistic adults were observed while playing video games together. Dialogue in each interaction was rated in terms of its logical coherence (e.g. how turns of dialogue are connected to each other), rapport (e.g. alignment in complimenting/supporting each other) and symmetry (e.g. how similar turn of dialogue are in terms of the way they are spoken, such as volume or length). To minimise the risk of interpreting interactions via normative social expectations (i.e., what non-autistic individuals perceive to be the 'right' way to interact), the study focussed on changes within interaction and involved autistic and non-autistic raters. The findings showed that moments of understanding could be achieved unconventionally in autistic adults. Aspects of interaction that non-autistic people might strive to avoid (e.g. not always reciprocating utterances, engaging in dialogue in parallel) could be beneficial as participants moved on from disruptive moments swiftly and were free to explore their own individual experiences during collaboration (which contributed to their making sense of the situation). Also participants made very generous assumptions of common ground that could lead to rapid rapport and humour when reciprocated. The study therefore highlights unrealised potential for achieving shared understanding among autistic adults and suggests that future research explore in greater detail how such potential could be enabled to flourish beyond autistic to autistic encounters.
