# Supplementary material for: Neurodivergent intersubjectivity: Distinctive features of how autistic people create shared understanding
Source: Autism. 2018 Aug 3;23(4):910–21. doi: 10.1177/1362361318785172 (PMC6512057; doi:10.1177/1362361318785172)
Supplement: Supplementary material [file AUT785172_Supplementary_file_1.pdf]

Supplementary file 1

Table 2
Transcription Conventions adapted from (Markee, 2015)

.	Period indicates a falling, or final, intonation contour, not necessarily the end of a sentence.
?	Question mark indicates rising intonation, not necessarily a question.
,	Comma indicates “continuing” intonation, not necessarily a clause boundary.
↑↓	Upward and downward pointing arrows indicate marked rising and falling shifts in intonation
:::	Colons indicate stretching of the preceding sound, proportional to the number of colons
-	A hyphen after a word or a part of a word indicates a cut-off or self-interruption with level pitch
word	Underlining indicates stress or emphasis.
WOrd	Upper case indicates loudness.
°word°	Degree signs enclose whispered speech
=	Equal sign indicate no break or delay between the words thereby connected.
<word>	Indicates slowed down delivery relative to surrounding talk
>word<	Indicates speeded up delivery relative to surrounding talk
(())	Double parentheses enclose descriptions of conduct.
(word)	When all or part of an utterance is in parentheses, this indicates uncertainty on the transcriber’s part.
()	Empty parentheses indicate that something is being said, but no hearing can be achieved.
(1.2)	Numbers in parentheses indicate silence in tenths of a second.
(.)	A dot in parentheses indicated a “micropause,” hearable but not readily measurable.
[	Separate left square brackets, one above the other on two successive lines with utterances by different speakers, indicates a point of overlap onset.
]	Separate right square brackets, one above the other on two successive lines with utterances by different speakers, indicates a point of overlap ending.
…	Ellipsis
(-)	Indicates unintelligible speech, each dash pertains to a syllable.

Markee, N. (2015). Appendix Transcription Conventions in Conversation Analysis. *The Handbook of Classroom Discourse and Interaction*, 527–528. http://doi.org/10.1002/9781118531242.app1
