# Supplementary material for: Neurodivergent intersubjectivity: Distinctive features of how autistic people create shared understanding
Source: Autism. 2018 Aug 3;23(4):910–21. doi: 10.1177/1362361318785172 (PMC6512057; doi:10.1177/1362361318785172)
Supplement: Supplementary material [file AUT785172_Supplementary_file_2.pdf]

Supplementary file 2

Within-interaction rating framework (W-IRF)

**Category**	**Rating definitions and indicators**		
Rating Values	-1	0	1
**Coherence**<br><br>Does the turn respond to the topic of the previous turn?	**Definition:** Fragmented from prior turn.<br>**Key indicators:**<br>(1) Ignores previous turn<br>(2) Sudden topic shift<br>(3) Interruptions<br>(4) Refusing to interact	**Definition:** Partial and ambiguous responses.<br>**Key indicators:**<br>(1) Any turn that is not clearly 'consistent' or 'fragmented'<br>(2) Unintelligible speech<br>(3) Yes/no answers to open questions<br>(4) Statements addressed to self<br>(5) Statement addressed to characters in the game	**Definition:** Consistent with prior turn.<br>**Key indicators:**<br>(1) Answering questions comprehensively, yes/no answers to closed questions<br>(2) "OK" to affirm action<br>(3) Continuing topic of discussion<br>(4) Etiquette greetings/goodbye<br>(5) Repetition of phrases or sounds made by partner
**Affect**<br><br>What type of emotion is displayed?	**Definition:** Negative affect.<br><br>**Key indicators:**<br>(1) Extreme negative comments<br>(2) Criticism<br>(3) Frustration<br>(4) Anger / Complaining	**Definition:** Affect ambiguous or not displayed.<br>**Key indicators:**<br>(1) Any turn that is not clearly 'negative or 'positive' affect	**Definition:** Positive affect.<br><br>**Key indicators:**<br>(1) Laughter<br>(2) Complimenting<br>(3) Encouraging<br>(4) Supporting
**Symmetry**<br><br>How assertive/submissive is the turn relative to prior turn?	**Definition:** Submissive.<br><br>**Key indicators:**<br>(1) Hesitation<br>(2) Turn interrupted<br>(3) Short turns in comparison to partner	**Definition:** Assertive/submissiveness ambiguous or not displayed.<br>**Key indicators:**<br>(1) Any turn that is not clearly 'assertive' or 'submissive'	**Definition:** Assertive.<br><br>**Key indicators:**<br>(1) Shouting<br>(2) Interrupting<br>(3) Imperatives<br>(4) Very long turns in comparison to partner
Note. Our goal was to understand within-interaction variability, and the constructs of coherence and symmetry examine alignment with respect to the prior turn. However, affect is operationalised in terms of harmony, because using alignment would result in an inflated affect score, because interpreting affect is dependent on observable features which may not always be possible to determine in each turn, thus leading to many 0 scores.			
