# Supplementary material for: Neurodivergent intersubjectivity: Distinctive features of how autistic people create shared understanding
Source: Autism. 2018 Aug 3;23(4):910–21. doi: 10.1177/1362361318785172 (PMC6512057; doi:10.1177/1362361318785172)

Supplementary file 3

Table 3

Mean and standard deviations of intersubjective dimensions

Interaction	Coherence Mean (S.D.)	Affect Mean (S.D.)	Symmetry Mean (S.D.)
1	0.23 (0.91)	0.25 (0.64)	0.33 (0.67)
2	0.18 (0.90)	0.41 (0.60)	0.48 (0.55)
3	0.49 (0.79)	0.16 (0.54)	0.35 (0.63)
4	0.36 (0.85)	-0.14 (0.65)	0.11 (0.75)
5	0.31 (0.88)	0.09 (0.61)	0.25 (0.65)
6	0.34 (0.87)	0.08 (0.47)	0.44 (0.65)
7	0.31 (0.93)	0.24 (0.51)	0.35 (0.66)
8	0.19 (0.83)	0.04 (0.58)	0.13 (0.67)
9	0.22 (0.93)	0.15 (0.60)	0.26 (0.68)
10	0.13 0.90)	0.09 (0.67)	0.48 (0.60)
11	0.48 (0.82)	0.36 (0.56)	0.53 (0.57)
12	0.44 (0.82)	0.25 (0.49)	0.46 (0.63)
13	0.44 (0.68)	0.33 (0.47)	0.35 (0.60)
14	0.33 (0.85)	0.41 (0.60)	0.36 (0.59)
15	0.34 (0.51)	0.50 (0.43)	0.18 (0.57)
16	0.39 (0.60)	0.38 (0.46)	0.39 (0.55)
17	0.45 (0.81)	0.16 (0.45)	0.39 (0.62)
18	0.41 (0.87)	0.37 (0.51)	0.42 (0.58)
19	0.41 (0.79)	0.23 (0.50)	0.21 (0.70)
20	0.24 (0.86)	0.36 (0.60)	0.25 (0.66)

Table 4

20-turn average highs and lows

Interaction	Coherence		Affect		Symmetry	
	Min	Max	Min	Max	Min	Max
1	-0.30	0.70	-0.30	0.65	-0.30	0.70
2	-0.45	0.72	-0.46	0.70	0.05	1.00
3	-0.20	1.00	-0.28	0.55	0.05	0.85
4	-0.38	0.94	-0.39	0.45	-0.45	0.56
5	-0.40	0.75	-0.39	0.60	-0.16	0.70
6	-0.16	0.90	-0.23	0.40	0.00	0.80
7	-0.17	1.00	-0.05	0.59	0.10	0.91
8	-0.18	0.64	-0.58	0.47	-0.13	0.47
9	-0.50	0.80	-0.26	0.80	-0.20	0.80
10	-0.55	1.00	-0.78	0.63	0.00	0.79
11	-0.21	1.00	0.00	0.76	0.00	0.81
12	-0.15	1.00	0.00	0.60	0.05	0.88
13	-0.11	1.00	0.00	0.68	-0.26	0.78
14	-0.27	0.87	-0.21	0.78	0.00	0.63
15	-0.10	0.75	-0.05	0.74	-0.20	0.55
16	-0.39	0.75	0.05	0.60	-0.35	0.80
17	-0.15	1.00	-0.13	0.60	0.05	0.80
18	-0.20	0.85	0.00	0.75	-0.08	0.65
19	-0.10	0.80	-0.05	0.50	-0.75	0.80
20	-0.54	0.94	-0.25	0.77	-0.41	0.82

Note. Across all interactions, all dimensions had 20 turns with a max score greater than +0.4 and a min score lower than +0.1.

Figure 3

Mean scores for coordination dimensions (with standard deviation bars around the mean)

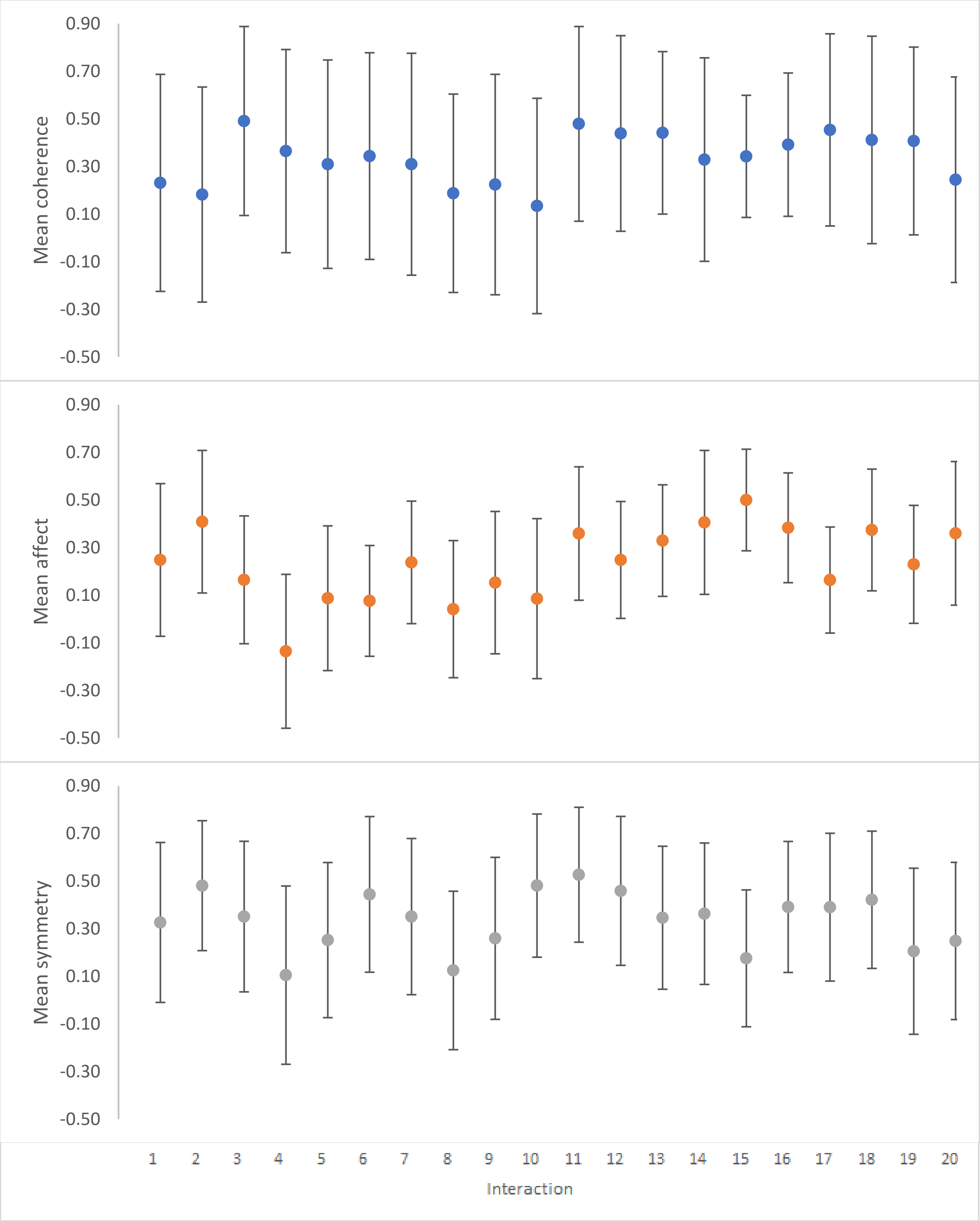

Supplement: Supplementary material [file AUT785172_Supplementary_file_3.pdf]
