# Supplementary material for: Neurodivergent intersubjectivity: Distinctive features of how autistic people create shared understanding
Source: Autism. 2018 Aug 3;23(4):910–21. doi: 10.1177/1362361318785172 (PMC6512057; doi:10.1177/1362361318785172)
Supplement: Supplementary material [file AUT785172_Supplementary_file_4.pdf]

Supplementary file 4

Table 5

Frequency of different voices (i.e. when the players assume a voice markedly different from their own) in the data sampled.

Voice type	Frequency	Reciprocated*	% Reciprocated
Player's avatar	115	81	70%
Fictional voices (TV/film/comics	50	26	52%
Accents/dialects	33	13	39%
Game AI	24	15	63%
unknown voice	24	13	54%
Narrator	19	12	63%
Miscellaneous**	19	14	73%
Social voices (other players/family)	12	10	83%
Music lyric	12	4	33%
Total	308	188	

*Reciprocated = instances where voices, in the next turn, are responded to in terms of coherence, affect or symmetry.

**Miscellaneous = Instances of voices that were very infrequent and did not belong to any other categories. These included idioms, anthropomorphising the voice of the Xbox console itself, sound effects and musical jingles.

Figure 4

Distribution of voicing across interactions*

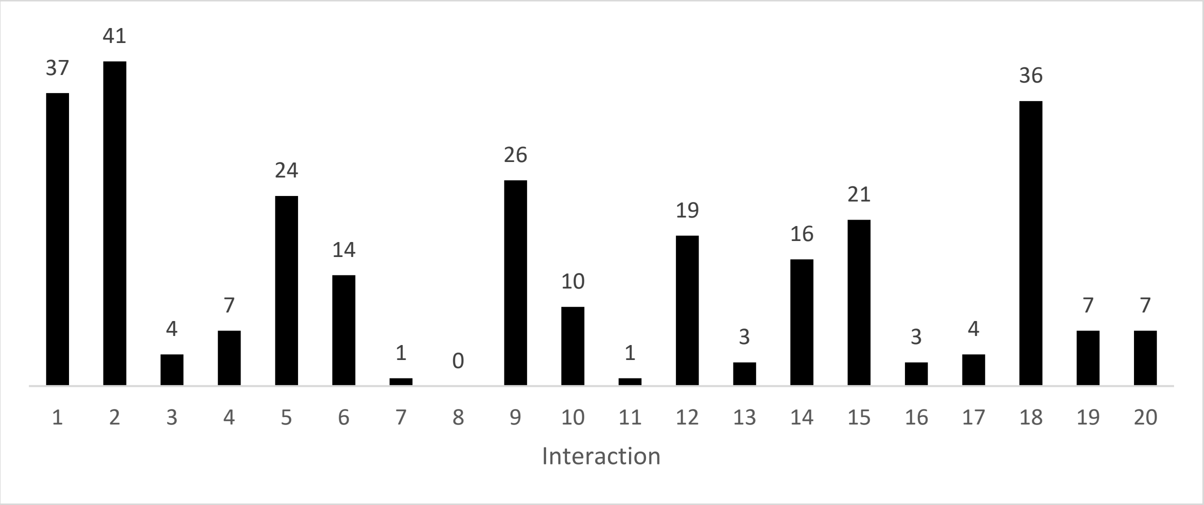

*This figure shows the distribution of voicing frequency (i.e. when the players assume a voice markedly different from their own) across all interactions.
